# Methyl 2-amino-5-bromo­benzoate

**DOI:** 10.1107/S1600536811025165

**Published:** 2011-07-02

**Authors:** Islam Ullah Khan, Muneeb Hayat Khan, Mehmet Akkurt

**Affiliations:** a Materials Chemistry Laboratory, Department of Chemistry, GC University, Lahore 54000, Pakistan; b Department of Physics, Faculty of Sciences, Erciyes University, 38039 Kayseri, Turkey

## Abstract

In the title compound, C_8_H_8_BrNO_2_, the dihedral angle between the aromatic ring and the methyl acetate side chain is 5.73 (12)°. The mol­ecular conformation is stabilized by an intra­molecular N—H⋯O hydrogen bond, generating an *S*(6) ring. In the crystal, mol­ecules are connected by N—H⋯O inter­actions, generating zigzag chains running along the *b*-axis direction.

## Related literature

For graph-set notation, see: Bernstein *et al.* (1995 ▶).
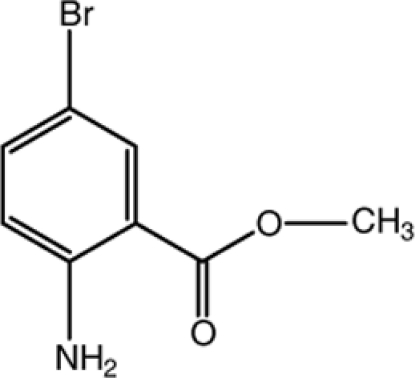


## Experimental

### 

#### Crystal data

C_8_H_8_BrNO_2_
*M* *_r_* = 230.05
Monoclinic, 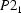

*a* = 3.9852 (2) Å
*b* = 9.1078 (5) Å
*c* = 12.1409 (7) Å
β = 95.238 (3)°
*V* = 438.83 (4) Å^3^
*Z* = 2
Mo *K*α radiation
μ = 4.64 mm^−1^
*T* = 296 K
0.21 × 0.19 × 0.15 mm

#### Data collection

Bruker APEXII CCD diffractometer
3878 measured reflections
1822 independent reflections
1570 reflections with *I* > 2σ(*I*)
*R*_int_ = 0.021

#### Refinement

*R*[*F* ^2^ > 2σ(*F* ^2^)] = 0.023
*wR*(*F* ^2^) = 0.045
*S* = 0.94
1822 reflections
116 parameters
3 restraints
H atoms treated by a mixture of independent and constrained refinement
Δρ_max_ = 0.29 e Å^−3^
Δρ_min_ = −0.24 e Å^−3^
Absolute structure: Flack (1983 ▶), 655 Freidel pairs
Flack parameter: 0.022 (9)

### 

Data collection: *APEX2* (Bruker, 2007 ▶); cell refinement: *SAINT* (Bruker, 2007 ▶); data reduction: *SAINT*; program(s) used to solve structure: *SHELXS97* (Sheldrick, 2008 ▶); program(s) used to refine structure: *SHELXL97* (Sheldrick, 2008 ▶); molecular graphics: *ORTEP-3 for Windows* (Farrugia, 1997 ▶); software used to prepare material for publication: *WinGX* (Farrugia, 1999 ▶) and *PLATON* (Spek, 2009 ▶).

## Supplementary Material

Crystal structure: contains datablock(s) global, I. DOI: 10.1107/S1600536811025165/hb5930sup1.cif

Structure factors: contains datablock(s) I. DOI: 10.1107/S1600536811025165/hb5930Isup2.hkl

Supplementary material file. DOI: 10.1107/S1600536811025165/hb5930Isup3.cml

Additional supplementary materials:  crystallographic information; 3D view; checkCIF report

## Figures and Tables

**Table 1:** Hydrogen-bond geometry (Å, °)

*D*—H⋯*A*	*D*—H	H⋯*A*	*D*⋯*A*	*D*—H⋯*A*
N1—H1*N*⋯O1	0.84 (2)	2.08 (3)	2.717 (3)	133 (3)
N1—H2*N*⋯O1^i^	0.85 (2)	2.22 (3)	3.039 (3)	162 (2)

Symmetry code: (i) 
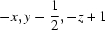
.

**Fig. 1.**
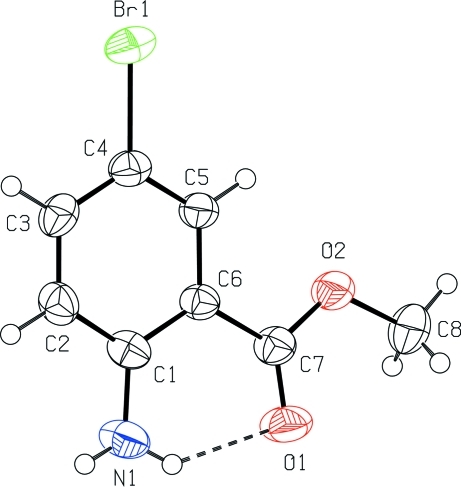
The title molecule with displacement ellipsoids for non-H atoms drawn at the 50% probability level.

**Fig. 2.**
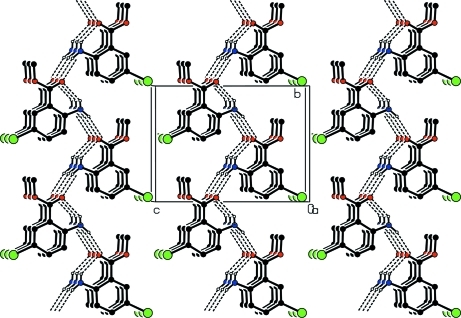
The packing and hydrogen bonding of the title compound viewing down the a axis. Hydrogen atoms not involved in hydrogen bonding have been omitted for clarity.

**Fig. 3.**
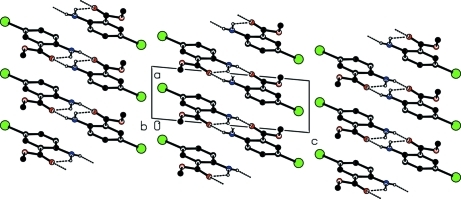
The packing and hydrogen bonding of the title compound viewing down the b axis. Hydrogen atoms not involved in hydrogen bonding have been omitted for clarity.

## supplementary crystallographic information

### Comment

The title compound is an intermediate for the synthesis of benzothiazines.

In the title compound (I), (Fig. 1), except H atoms, all atoms are almost coplanar with the maximum deviations of -0.122 (2) Å for O1, 0.077 (2) Å for O2, -0.050 (1) Å for Br1 and 0.048 (3) Å for C8. The C5—C6—C7—O1, C5—C6—C7—O2, C6—C7—O2—C8 and O1—C7—O2—C8 torsion angles are 174.3 (2), -5.2 (3), -178.9 (2) and 0.6 (3)°, respectively.

The molecular conformation is stabilized by intramolecular N—H···O hydrogen bond (Table 1, Fig. 1) forming S(6) ring (Bernstein *et al.*, 1995). In the crystal structure, intermolecular N—H···O interactions link the neighbouring molecules to each other, producing a zigzag chain along the *b* axis (Table 1). Figures 2 and 3 show the packing and hydrogen bonding of the title compound viewing down the a and b-axes, respectively.

### Experimental

The title compound was purchased from Sigma-Aldrich and recrystalized in methanol to yield light yellow blocks.

### Refinement

In the last cycles of the refinement, 3 reflections (0 1 1), (0 - 1 1) and (0 0 1) were eliminated due to being poorly measured in the vicinity of the beam stop. The H atoms of the NH_2_ group were located in a difference Fourier map, and refined with a distance restraint N—H = 0.86 (2) Å. Their isotropic displacement parameters were set to be 1.2*U*_eq_(N). The other H atoms were positioned geometrically with C–H distances of 0.93 Å (aromatic) and 0.96 Å (methyl), and refined using a riding model, with *U*_iso_(H) = 1.2*U*_eq_(C) for aromatic and *U*_iso_(H) = 1.5*U*_eq_(C) for methyl.

### Crystal data

**Table:**

C_8_H_8_BrNO_2_	*F*(000) = 228
*M**_r_* = 230.05	*D*_x_ = 1.741 Mg m^−^^3^
Monoclinic, *P*2_1_	Mo *K*α radiation, λ = 0.71073 Å
Hall symbol: P 2yb	Cell parameters from 2303 reflections
*a* = 3.9852 (2) Å	θ = 2.8–26.3°
*b* = 9.1078 (5) Å	µ = 4.64 mm^−^^1^
*c* = 12.1409 (7) Å	*T* = 296 K
β = 95.238 (3)°	Block, light yellow
*V* = 438.83 (4) Å^3^	0.21 × 0.19 × 0.15 mm
*Z* = 2	

### Data collection

**Table:**

Bruker APEXII CCD diffractometer	1570 reflections with *I* > 2σ(*I*)
Radiation source: sealed tube	*R*_int_ = 0.021
graphite	θ_max_ = 28.3°, θ_min_ = 3.4°
φ and ω scans	*h* = −3→5
3878 measured reflections	*k* = −12→10
1822 independent reflections	*l* = −15→15

### Refinement

**Table:**

Refinement on *F*^2^	Secondary atom site location: difference Fourier map
Least-squares matrix: full	Hydrogen site location: inferred from neighbouring sites
*R*[*F*^2^ > 2σ(*F*^2^)] = 0.023	H atoms treated by a mixture of independent and constrained refinement
*wR*(*F*^2^) = 0.045	*w* = 1/[σ^2^(*F*_o_^2^) + (0.*P*)^2^] where *P* = (*F*_o_^2^ + 2*F*_c_^2^)/3
*S* = 0.94	(Δ/σ)_max_ < 0.001
1822 reflections	Δρ_max_ = 0.29 e Å^−^^3^
116 parameters	Δρ_min_ = −0.24 e Å^−^^3^
3 restraints	Absolute structure: Flack (1983), 655 Freidel pairs
Primary atom site location: structure-invariant direct methods	Flack parameter: 0.022 (9)

### Special details

**Table:**

Geometry. Bond distances, angles *etc*. have been calculated using the rounded fractional coordinates. All su's are estimated from the variances of the (full) variance-covariance matrix. The cell e.s.d.'s are taken into account in the estimation of distances, angles and torsion angles
Refinement. Refinement on *F*^2^ for ALL reflections except those flagged by the user for potential systematic errors. Weighted *R*-factors *wR* and all goodnesses of fit *S* are based on *F*^2^, conventional *R*-factors *R* are based on *F*, with *F* set to zero for negative *F*^2^. The observed criterion of *F*^2^ > σ(*F*^2^) is used only for calculating -*R*-factor-obs *etc*. and is not relevant to the choice of reflections for refinement. *R*-factors based on *F*^2^ are statistically about twice as large as those based on *F*, and *R*-factors based on ALL data will be even larger.

### Fractional atomic coordinates and isotropic or equivalent isotropic displacement parameters (Å^2^)

**Table:**

	*x*	*y*	*z*	*U*_iso_*/*U*_eq_	
Br1	0.60353 (6)	0.04286 (5)	0.06725 (2)	0.0596 (1)	
O1	−0.0244 (5)	0.5400 (3)	0.36497 (11)	0.0619 (6)	
O2	0.1200 (4)	0.5450 (3)	0.19255 (11)	0.0510 (5)	
N1	0.1618 (6)	0.3011 (2)	0.48993 (17)	0.0516 (8)	
C1	0.2591 (6)	0.2478 (2)	0.39185 (18)	0.0377 (8)	
C2	0.3968 (6)	0.1064 (2)	0.3901 (2)	0.0434 (8)	
C3	0.4990 (5)	0.0477 (4)	0.29613 (16)	0.0435 (7)	
C4	0.4676 (6)	0.1276 (2)	0.19848 (18)	0.0403 (8)	
C5	0.3422 (6)	0.2668 (2)	0.19724 (18)	0.0378 (7)	
C6	0.2343 (6)	0.3290 (2)	0.29357 (17)	0.0348 (7)	
C7	0.0988 (6)	0.4787 (2)	0.28974 (19)	0.0394 (8)	
C8	−0.0137 (8)	0.6915 (3)	0.1821 (2)	0.0596 (10)	
H1N	0.039 (8)	0.376 (2)	0.484 (3)	0.0710*	
H2	0.41870	0.05150	0.45500	0.0520*	
H2N	0.147 (7)	0.240 (3)	0.5425 (19)	0.0710*	
H3	0.59020	−0.04640	0.29710	0.0520*	
H5	0.32810	0.32100	0.13200	0.0450*	
H8A	−0.24790	0.69040	0.19480	0.0900*	
H8B	0.01050	0.72780	0.10900	0.0900*	
H8C	0.10720	0.75420	0.23550	0.0900*	

### Atomic displacement parameters (Å^2^)

**Table:**

	*U*^11^	*U*^22^	*U*^33^	*U*^12^	*U*^13^	*U*^23^
Br1	0.0709 (2)	0.0566 (2)	0.0534 (2)	0.0159 (2)	0.0178 (1)	−0.0131 (2)
O1	0.1002 (13)	0.0407 (8)	0.0489 (9)	0.0161 (15)	0.0288 (8)	−0.0031 (12)
O2	0.0743 (10)	0.0373 (8)	0.0435 (8)	0.0152 (14)	0.0164 (7)	0.0039 (11)
N1	0.0771 (17)	0.0435 (13)	0.0359 (12)	−0.0050 (11)	0.0151 (11)	−0.0012 (10)
C1	0.0446 (14)	0.0347 (13)	0.0338 (12)	−0.0104 (10)	0.0036 (10)	−0.0033 (10)
C2	0.0526 (16)	0.0379 (13)	0.0396 (13)	−0.0011 (10)	0.0042 (10)	0.0078 (10)
C3	0.0469 (12)	0.0305 (11)	0.0528 (12)	0.0054 (16)	0.0035 (9)	−0.0033 (17)
C4	0.0432 (14)	0.0403 (14)	0.0378 (13)	0.0010 (10)	0.0059 (10)	−0.0076 (10)
C5	0.0468 (14)	0.0342 (12)	0.0333 (12)	0.0032 (10)	0.0089 (9)	0.0008 (10)
C6	0.0397 (14)	0.0317 (12)	0.0334 (12)	−0.0024 (9)	0.0050 (9)	−0.0032 (9)
C7	0.0482 (15)	0.0304 (11)	0.0400 (13)	−0.0016 (9)	0.0058 (10)	−0.0035 (10)
C8	0.080 (2)	0.0332 (14)	0.0659 (18)	0.0172 (13)	0.0088 (14)	0.0075 (12)

### Geometric parameters (Å, °)

**Table:**

Br1—C4	1.893 (2)	C3—C4	1.387 (3)
O1—C7	1.212 (3)	C4—C5	1.362 (3)
O2—C7	1.335 (3)	C5—C6	1.402 (3)
O2—C8	1.438 (4)	C6—C7	1.466 (3)
N1—C1	1.374 (3)	C2—H2	0.9300
N1—H1N	0.84 (2)	C3—H3	0.9300
N1—H2N	0.85 (2)	C5—H5	0.9300
C1—C6	1.400 (3)	C8—H8A	0.9600
C1—C2	1.401 (3)	C8—H8B	0.9600
C2—C3	1.356 (3)	C8—H8C	0.9600
			
C7—O2—C8	116.41 (18)	O1—C7—O2	121.4 (2)
C1—N1—H1N	115 (2)	O1—C7—C6	125.3 (2)
C1—N1—H2N	117.5 (18)	O2—C7—C6	113.3 (2)
H1N—N1—H2N	121 (3)	C1—C2—H2	119.00
C2—C1—C6	118.0 (2)	C3—C2—H2	119.00
N1—C1—C2	118.68 (19)	C2—C3—H3	120.00
N1—C1—C6	123.27 (18)	C4—C3—H3	120.00
C1—C2—C3	121.6 (2)	C4—C5—H5	120.00
C2—C3—C4	120.1 (3)	C6—C5—H5	120.00
Br1—C4—C3	119.67 (17)	O2—C8—H8A	110.00
Br1—C4—C5	120.19 (16)	O2—C8—H8B	109.00
C3—C4—C5	120.1 (2)	O2—C8—H8C	109.00
C4—C5—C6	120.46 (19)	H8A—C8—H8B	109.00
C5—C6—C7	119.32 (18)	H8A—C8—H8C	109.00
C1—C6—C5	119.64 (18)	H8B—C8—H8C	109.00
C1—C6—C7	121.05 (19)		
			
C8—O2—C7—C6	178.9 (2)	C2—C3—C4—C5	1.7 (4)
C8—O2—C7—O1	−0.6 (3)	Br1—C4—C5—C6	178.61 (18)
C6—C1—C2—C3	−1.1 (3)	C3—C4—C5—C6	−1.9 (4)
N1—C1—C2—C3	−179.7 (2)	C4—C5—C6—C7	−179.7 (2)
C2—C1—C6—C5	0.8 (3)	C4—C5—C6—C1	0.7 (3)
C2—C1—C6—C7	−178.8 (2)	C1—C6—C7—O1	−6.0 (4)
N1—C1—C6—C5	179.4 (2)	C5—C6—C7—O2	−5.2 (3)
N1—C1—C6—C7	−0.2 (4)	C1—C6—C7—O2	174.5 (2)
C1—C2—C3—C4	−0.2 (4)	C5—C6—C7—O1	174.3 (2)
C2—C3—C4—Br1	−178.82 (18)		

### Hydrogen-bond geometry (Å, °)

**Table:**

*D*—H···*A*	*D*—H	H···*A*	*D*···*A*	*D*—H···*A*
N1—H1N···O1	0.84 (2)	2.08 (3)	2.717 (3)	133 (3)
N1—H2N···O1^i^	0.85 (2)	2.22 (3)	3.039 (3)	162 (2)

Symmetry codes: (i) −*x*, *y*−1/2, −*z*+1.
